# Uptake of *Levilactobacillus brevis* JCM 1059 by THP-1 Cells via Interaction between SlpB and CAP-1 Promotes Cytokine Production

**DOI:** 10.3390/microorganisms11020247

**Published:** 2023-01-18

**Authors:** Tingyu Yin, Xiaoxi Zhang, Shun Iwatani, Kazuhiko Miyanaga, Naoyuki Yamamoto

**Affiliations:** 1 School of Life Science and Technology, Tokyo Institute of Technology, Yokohama 226-8501, Kanagawa, Japan; 2 Department of Microbiology and Immunology, School of Medicine, Keio University, 35 Shinanomachi, Shinjuku, Tokyo 160-8582, Japan; 3 Tsukuba Biotechnology Research Center, Astellas Pharma Inc., 5-2-3, Tokodai, Tsukuba-shi 300-2698, Ibaraki, Japan; 4 Department of Infection and Immunity, School of Medicine, Jichi Medical University, 3311-1, Yakushiji, Shimotsuke-shi 329-0498, Tochigi, Japan

**Keywords:** lactic acid bacteria, THP-1, dendritic cells, *Levilactobacillus brevis*, SlpB, CAP-1, IL-12

## Abstract

Several probiotic lactic acid bacteria (LAB) exert immunomodulatory effects on the host. However, the reasons for the different effects of LAB have not been fully elucidated. To understand the different immunomodulatory effects of LAB, we evaluated the levels of critical molecules in differentiated monocytic THP-1 and dendritic cells (DCs) following the uptake of various LAB strains. *Lactobacillus helveticus* JCM 1120, *Lactobacillus acidophilus* JCM 1132, *Levilactobacillus brevis* JCM 1059, and *Lentilactobacillus kefiri* JCM 5818 showed significantly higher uptake among the 12 LAB species tested. The uptake of microbeads by THP-1 DC increased when coupled with the surface layer proteins (Slps) from the tested strains. SlpB was mainly observed in the *L. brevis* JCM 1059 Slps extract. The expected cell surface receptor for SlpB on THP-1 DC was purified using SlpB-coupled affinity resin and identified as adenylyl cyclase-associated protein 1 (CAP-1). SlpB binding to THP-1 DC decreased after the addition of anti-CAP-1 and anti-DC-SIGN antibodies but not after the addition of anti-macrophage-inducible C-type lectin (Mincle) antibody. These results suggest that SlpB on *L. brevis* JCM 1059 plays preferentially binds to CAP-1 on THP-1 DC and plays a crucial role in bacterial uptake by THP-1 cells as well as in subsequent interleukin-12 (IL-12) production.

## 1. Introduction

Probiotics are bioactive microbes that have beneficial effects on the host and improve their intestinal microbial balance [1]. The human microbiota primarily includes bacteria that can profoundly influence health and disease. Several studies have shown that probiotics have various biological functions. They can improve intestinal morphology, maintain intestinal microbial balance, and improve host immunity [2,3]. Several probiotic lactic acid bacteria (LAB) exert immunomodulatory effects on the host, protecting from the infections caused by pathogenic bacteria and suppressing allergic symptoms [4]. *Lactobacillus* strains are known to be potent inducers of proinflammatory cytokines, such as interleukin-12 (IL-12) and tumour necrosis factor α (TNFα) in the gut [5,6]. The immunomodulatory effects of lactobacilli are closely linked to their uptake by the gut-associated lymphoid tissue (GALT) and ability to modulate mucosal immune responses. Sampling intestinal bacteria, such as probiotic LAB strains, by the mucosal epithelium is essential for initiating immune responses in the GALT. The uptake of LAB by the microfold cells (M cells) in the follicle-associated epithelium (FAE) is a crucial event for the activation of the immune cells, such as the antigen-presenting dendritic cells (DCs), within the lymphoid follicles of the GALT [7].

DCs play a crucial role in defence against many pathogens by inducing cellular immunity after pathogen recognition [8,9]. DCs are activated in response to intestinal microbes and mediate the differentiation of naïve T cells into T helper type 1 (Th1) and Th2 cells [10]. DCs recognise pathogens via cell surface receptors, such as toll-like receptors [11,12] and C-type lectins [13]. DC-specific ICAM-3-grabbing non-integrin (DC-SIGN) is a DC-specific type II transmembrane protein with a C-type lectin extracellular domain [14,15] that plays a crucial role in the first contact between DCs, and probiotic and pathogenic bacteria.

Microbial-associated molecular patterns (MAMPs) modulate multiple host immune responses. Lipopolysaccharide (LPS), lipoproteins (LP), peptidoglycan (PG), polysaccharide A (PSA), lipoteichoic acids (LTA), microbial RNA, and DNA have been reported as MAMPs components [16]. Recognition of pathogenic and commensal bacteria-derived MAMPs by host pattern recognition receptors (PRRs) leads to the induction of different host immune responses [17].

*Enterobacteriaceae*, such as *Escherichia coli* [18], *Shigella* spp., and *Salmonella* spp [19,20] have been reported to induce phagocytosis after binding to DC-SIGN on DC surfaces. Among LAB, the surface layer proteins (Slps) on *Lactobacillus acidophilus* [21,22], *Lentilactobacillus kefiri* [23], and *Lactobacillus helveticus* [22] have been shown to bind to the DC-SIGN receptor induced on DCs. Macrophage-inducible C-type lectin (Mincle), a C-type lectin on differentiated macrophages, was reported to be the receptor for the Slps from *Levilactobacillus brevis* [24]. However, the binding of Slps of *Lactobacillus* species with various sequences and isoelectric points [25] involved in these immunomodulatory properties remains unknown. Slps are readily released from *Lactobacillus* cells by treatment with chaotropic reagents, such as LiCl [26], due to their non-covalent ionic binding to the cell surface. Twelve kinds of strains commonly used as probiotic LAB species were randomly selected. The major Slps from various LAB strains were coupled with microbeads to determine their ability to support bacterial uptake. Additionally, the critical receptors on THP-1 DC, which are involved in LAB binding, were identified.

Here, we report a novel receptor protein called adenylyl cyclase-associated protein 1(CAP-1), which is expressed on THP-1 DC and has an affinity for *L. brevis* JCM 1059 SlpB. Finally, we outline the importance of SlpB-CAP-1 binding for cellular uptake and subsequent cytokine production.

## 2. Materials and Methods

### 2.1. Bacterial Strains and Fermentation

All LAB strains listed in the Table 1 were obtained from the Japan Collection of Microorganisms (JCM) and our culture collections. LAB strains were cultured in Man Rogosa Sharpe (MRS, Becton, Dickinson and Company, Sparks, MD, USA) medium at 30 °C or 37 °C for 20 h, as described in Table 1. Human originated THP-1 monocyte cells were obtained from RIKEN Cell Bank (JRCB0112).

### 2.2. Induction of DC-SIGN and CAP-1 Expression on Differentiated THP-1 Cells

To confirm the differentiation of THP-1 cells into DC (THP-1 DC), the cell surface expression of DC-SIGN was evaluated by flow cytometry (EC800, SONY) after adding anti-DC-SIGN (Novus Biologicals USA, Centennial, CO, USA) and anti-CAP-1 antibodies (Novus Biologicals USA, Centennial, CO, USA). THP-1 cells obtained from JCM were maintained in Roswell Park Memorial Institute 1640 (RPMI 1640) supplemented with 10% fetal bovine serum (FBS) at 37 °C in a 5% CO_2_ humidified incubator. THP-1 cells were seeded in 24-well culture plates (3 × 10^5^ cells/mL) and treated with 50 nM phorbol 2-myristate 13-acetate (PMA, Adipogen Life Science, Liestal, Switzerland) and 20 ng/mL IL-4 (Peprotech, Cranbury, NJ, USA) to promote the differentiation of THP-1 DCs. THP-1 cells were cultured with PMA for 1 d, PMA mixed with IL-4 for one day, PMA for two days, PMA mixed with IL-4 for two days, or PMA for two days, followed by IL-4 for two days at 37 °C. Before harvesting the cells, they were incubated in 4% paraformaldehyde (PFA) for 10 min. The cells in each well were then detached by adding 1 mM ethylenediaminetetraacetic acid (EDTA)-PBS and transferred to a 1.5 mL plastic microtube. After washing the cells thrice with PBS, DC-SIGN and CAP-1 gene expression were monitored at 490/525 nm via flow cytometry. Anti-DC-SIGN or anti-CAP-1 antibody was added to the cells (5 ng/mL) before incubation at room temperature for 1 h. After washing the cells with PBS, they were mixed with anti-IgG-Alexa 488 (50 ng/mL, Thermo Fisher Scientific Company, Waltham, MA, USA) and incubated at 25 °C for 1 h.

### 2.3. Sulfo-Cyanine3 Labelling

Twelve types of LAB cells were labelled with sulfo-cyanine3 (Cy3) using a Cy3 Mono-reactive dye labelling kit (GE Healthcare Bio-Sciences KK, Tokyo, Japan), according to the manufacturer’s instructions. Briefly, all LAB strains were cultured in 50 mL MRS medium at 30 °C or 37 °C for 20 h, harvested via centrifugation at 6000× *g* for 10 min, and then washed twice with 5 mL of 0.1 M NaHCO_3_ (pH = 9.3). Cells were suspended in 500 μL of 1 M NaHCO_3_ (pH 9.3) and mixed with the Cy3-labelling reagent at 25 °C for 1 h. The Cy3 labelled LAB were washed with PBS and used for the THP-1 DC uptake study.

### 2.4. Preparation of Slps Coupled Microbeads

Twelve LAB strains were washed twicw with PBS by centrifugation at 6000× *g* for 10 min to collect the pellet. Then the cells in the pellet were suspended in 1 M LiCl and washed once with 1 M LiCl. Then, the collected cells were suspended in 20 mL of 5M LiCl and the supernatant was collected after centrifugation and dialysed against 300 times the volume of 20 mM phosphate buffer (pH 6.8) and freeze-dried for storage at −30 °C until future use. To prepare the Slps and ovalbumin conjugated fluorescein isothiocyanate (^FITC^OVA, Invitrogen) coupled microbeads, Slps (5 μg/mL) and ^FITC^OVA (5 μg/mL) were incubated with the hydrophilic 1-ethyl-3-(3-dimethylaminopropyl) carbodiimide hydrochloride (GN) microbeads (Kamakura Techno-Science, Inc., Tokyo, Japan) pre-activated in 3.3% (*v*/*v*) glutaraldehyde at 30 °C for 1 h (Slps-microbeads). Subsequently, the Slps-microbeads were blocked by mixing with 40 nM glycine-PBS (phosphate buffered saline, pH 7.4, 0.8% NaCl) at 30 °C for 1 h.

### 2.5. Uptake of LAB and Slps-Microbeads by THP-1 DC

To prepare THP-1 DC, THP-1 cells were suspended in RPMI 1640 containing 10% FBS, 50 nM PMA, and 20 ng/mL IL-4 and seeded on a 24-well culture plate at a concentration of 1 × 10^6^ cells/mL for two days at 37 °C. THP-1 DC were co-cultured with Cy3 labelled LAB cells (MOI = 10) for 2 h in 24-well culture plates with 500 μL RPMI 1640 containing 10% FBS. After cultivation, THP-1 DC were washed thrice with PBS and harvested by adding 10 mM EDTA containing PBS. Uptake of Cy3 labelled LAB and ^FITC^OVA was measured using a SONY EC800 flow cytometer. Flow cytometric analyses were performed at 550/570 and 490/525 nm.

### 2.6. Cytokine Measurement

THP-1 DC were cultured in RPMI 1640 medium supplemented with 10% FBS, 100 U/mL penicillin, and 100 μg/mL streptomycin. The cells were then stimulated in 96-well culture plates with 50 nM PMA for 1 or 2 d, with or without IL-4 (final concentration of 20 ng/mL). Cells in the other two groups were incubated with PMA for 1 or 2 d and additional 1 or 2 d with IL-4, respectively. Heat-killed Lactobacillus cells were incubated with THP-1 DC (MOI = 10) at 37 °C for 24 h. After incubation, the culture supernatant was collected by centrifugation at 6000× *g* for 10 min. IL-10 and IL-12p40 (IL-12) in the supernatant were analyzed by using ELISA kits obtained from BioLegend Inc. (San Diego, CA, USA) and R&D systems (Minneapolis, MN, USA), respectively, with a plate reader (Thermo Fisher Scientific, Varioskan LUX SkanIt Software 4.0) in triplicates.

### 2.7. Purification of the SlpB Receptor on THP-1 Cells

To purify the SlpB receptor on THP-1 cells, purified SlpB from L. brevis JCM 1059 was covalently coupled with an affinity resin. The affinity resin was prepared by mixing 10 mg purified JCM 1059 SlpB with 1 mL of Profinity Epoxide (Bio-Rad Laboratories, Inc. Hercules, CA, USA). THP-1 DC (differentiated with 50 nM PMA and 20 ng/mL IL-4 for 1 d) were washed with PBS and harvested. Subsequently, their cell surface components were extracted with 0.1% Triton PBS (cell extract). The cell extract was centrifuged at 8000× *g* for 10 min to remove aggregates and cell debris. After centrifugation, the supernatant was mixed with the SlpB-resin and incubated at room temperature for 1 h. Next, the SlpB-resin was washed with 0.15 M NaCl PB containing 0.1% Triton-X 100. Proteins with an affinity for SlpB were eluted by washing with 0.1% triton containing 0.5 and 1 M NaCl PB.

### 2.8. Sodium Dodecyl Sulphate-Polyacrylamide Gel Electrophoresis and Western Blotting Analysis

Slps released from the 12 LAB with the help of 5 M LiCl were analysed using sodium dodecyl sulphate-10% polyacrylamide gel electrophoresis (SDS-10%PAGE). The released proteins were mixed with sample buffer (6 × 125 nM Tris-HCl, 4% SDS, 20% glycerol, 0.012% bromophenol blue, and 10% 2-mercaptoethanol) and heated for 5 min at 95 °C. Then, the Slps were analysed by SDS-10%PAGE according to the Laemmli method [27]. Protein bands in the gel were visualised by staining the gels with Coomassie Brilliant Blue (CBB). Protein Ladder One Plus (Nacalai Tesque, Kyoto, Japan) was used as the marker. Proteins in the gel were transferred to polyvinylidene difluoride (PVDF) membranes in Tris-Cl buffer, pH 8.3 (190 mM glycine, 5 mM Tris-Cl, 20% methanol), for 1 h, at 150 mA. Specific proteins were detected with antibodies against Mincle (NK MAX, Santa Ana, CA, USA), DC-SIGN (SAB, Greenbelt, MD, USA), and CAP-1 (Novus Biological. Centennial, CO, USA) after 1000-fold dilution and following bionylated anti-rabbit IgG after 1000-fold dilution and avidin-peroxidase reaction). Finally, the band was detected by adding of 4-Chloro-1-naphthol as a substrate (Wako, Japan).

### 2.9. Protein Identification

Proteome analysis was performed to identify the proteins. Protein bands were excised from SDS-10%PAGE gels after CBB staining. To remove the dye from the gel, a de-staining solution (30% acetonitrile, 50 mM NH_4_HCO_3_) was added and incubated for 30 min. Then, 60% acetonitrile and 20 mM NH_4_HCO_3_ were added to remove water from the gel. Next, 5% (*w*/*w*) trypsin (Promega Japan, Tokyo, Japan) was added to the dried gel and incubated at 37 °C for 12 h. Peptides released from the gel were analysed by mass spectrometry using an UltrafleXtreme TOF/TOF MS (Bruker Daltonics GmbH, Bremen, Germany) operating in positive reflection ion mode between m/z 0 and 5000 Da.

### 2.10. Binding of Glycan and SlpB to THP-1

THP-1 DCs were washed twice with cold PBS after differentiation and were fixed in 1% PFA for 10 min at 4 °C. Subsequently, ^Cy3^SlpB was added (0.3 mg/mL) with or without galactose (Sigma-Aldrich, St. Louis, MO, USA) or mannose (Nacalai Tesque Inc., Kyoto, Japan) (each 0.5 mg/mL) and incubated for 1 h at 37 °C. THP-1 DCs were collected and subjected to flow cytometry to evaluate the binding of ^Cy3^SlpB to THP-1 DC.

### 2.11. Binding of Deglycosylated SlpB to THP-1

A microwell plate was coated with anti-CAP-1 antibody (1 µg/mL) for 24 h at 4 °C, and THP-1 DC cell extract was incubated for 2 h at 25 °C. Then, ^Cy3^SlpA or deglycosylated ^Cy3^SlpB were added to each well, and the plates were incubated for 2 h at 25 °C. To remove the polysaccharides from SlpB, 4 µg of recombinant glycosidase (PNGase F PRIME^TM^, N-Zyme Scientifics, Doylestown， PA, USA) was mixed with 20 µg of ^Cy3^SlpB in PBS and incubated for 24 h at 37 °C. Cy3 fluorescence originating from ^Cy3^SlpB was measured using a plate reader (Thermo Fisher Scientific, Varioskan LUX SkanIt Software 4.0).

### 2.12. Statistical Analysis

Statistical significance was analysed using GraphPad Prism software version 9.1. Statistically significant differences were was set at *p* < 0.05 by using one-way analysis of variance (ANOVA) followed by Duncan’s test.

## 3. Results

### 3.1. Induction of DC-SIGN on THP-1 Cells

**Figure 1:**
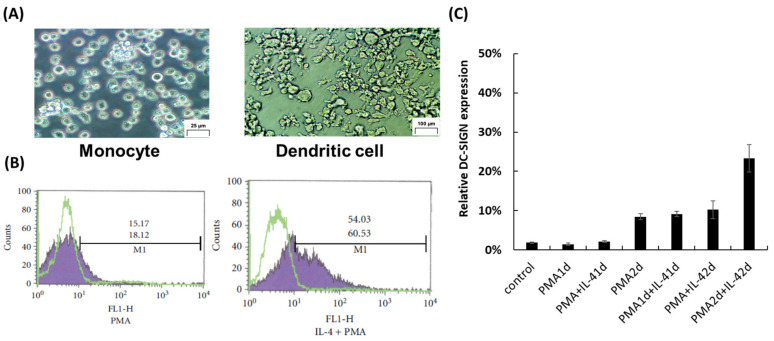
(**A**) Differentiation of monocytic THP-1 cells into dendritic-like THP-1 cells (THP-1 DC) by treatment with phorbol 2-myristate 13-acetate (PMA) and interleukin-4 (IL-4). (**B**) Expression of DC-specific ICAM-3-grabbing nonintegrin (DC-SIGN) on the surface of THP-1 DC was evaluated by flow cytometer using anti-DC-SIGN antibody. (**C**) Quantification of THP-1 cells expressing DC-SIGN on their cell surfaces after PMA and IL-4 treatment.

A previous study reported that SlpA binding was required for the basal level expression of DC-SIGN on THP-1 DC after PMA and IL-4 treatment [21,28,29]. Here, THP-1 cells were treated with a combination of PMA and IL-4 according to a previously report [29]. THP-1 cells showed a dendritic-like morphology (THP-1 DC) when PMA treatment (for 2 d) was followed by IL-4 treatment (for 2 d) (Figure 1A). The expression of DC-SIGN on THP-1 DC was then quantified as one of the differentiation markers by flow cytometry using anti-DC-SIGN antibody (Figure 1B). The results showed that administering PMA and PMA combined with IL-4 for 1 d was not effective in inducing DC-SIGN (induced less than 2%) expression (Figure 1C). Conversely, treatment with PMA for 2 d, PMA for 1 d and IL-4 for 1 d, or PMA combined with IL-4 for 2 d showed increased DC-SIGN expression on THP-1 cell surface (8.5, 9.2, and 10.3%, respectively) whereas the impact of additional IL-4 on DC-SIGN induction was not clear (Figure 1C). DC-SIGN levels on the cell surface were highest on THP-1 DC that were subjected to 2 days of PMA treatment, followed by IL-4 treatment for 2 days (23.3%) (Figure 1C).

### 3.2. Uptake of LAB by THP-1 DC and IL-12 Induction

**Figure 2:**
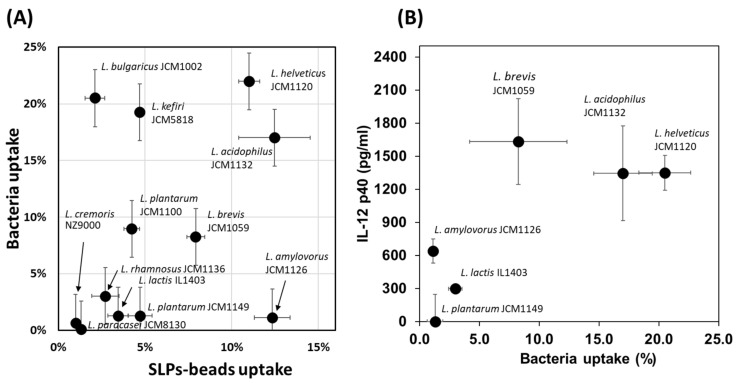
(**A**) Uptakes of Cy3 labelled surface layer protein A (^Cy3^SlpA) lactic acid bacteria (LAB) and Slps-coupled microbeads by THP-1 DC. (**B**) Uptake of Cy3 labelled LAB (Bacterial uptake), and IL-12 production from THP-1 cells by treatments with various LAB strains.

The SlpA on specific LAB strains is the key protein that binds to the DC-SIGN on THP-1 DC and plays a crucial role in the subsequent immune reaction. However, little is known about the other LAB cell surface proteins that may be capable of THP-1 DC binding. Twelve LAB strains were labelled with Cy3 and their uptake by THP-1 DC was analysed by flow cytometry. Significant differences were observed in the uptake ratios of the 12 LAB strains that were tested (Figure 2A). In particular, *L. helveticus* JCM 1120, *Lactobacillus delbrueckii* subsp. *bulgaricus* JCM 1002, *L. kefiri* JCM 5818, *L. acidophilus* JCM 1132, *Lactiplantibacillus plantarum* JCM 1100, and *L. brevis* JCM 1059 showed significantly higher uptake ratios than that of *Lacticaseibacillus paracasei* subsp. *paracasei* JCM 8130, which had the lowest uptake ratio (Figure 2A). Next, the Slps that were released from the LAB strains after treatment with the chaotropic reagent 5 M LiCl were coupled to ^FITC^OVA conjugated microbeads and the importance of Slps in the uptake of the microbeads by THP-1 DC was evaluated. The microbeads coupled with Slps from various LAB strains showed different uptake ratios among the tested strains (Figure 2A). Microbeads-coupled with the Slps from *L. helveticus* JCM 1120, *L. acidophilus* JCM 1132, *L. brevis* JCM 1059, *L. plantarum* JCM 1100, and *L. kefiri* JCM 5818 showed potent uptake ratios, whereas decreased uptake ratios were observed for the microbeads that were coupled with the Slps from the LAB strains exhibiting reduced uptake (Figure 2A). This strongly suggests that the Slps released from *L. helveticus* JCM 1120, *L. acidophilus* JCM 1132, *L. brevis* JCM 1059, *L. plantarum* JCM 1100, and *L. kefiri* JCM 5818 contain the key components necessary for the binding of the bacteria to specific receptors on THP-1 DC. To understand the effect of LAB uptake on cytokine production, we monitored IL-12 production in THP-1 DC after treatment with 6 different LAB strains. Among the tested 6 strains, two types of *L. lactis* subspecies with lower uptake ratios showed lower IL-12 production, whereas *L. helveticus* JCM 1120, *L. acidophilus* JCM 1132, and *L. brevis* JCM 1059 with higher uptake ratios showed higher IL-12 production (Figure 2B). Therefore, the increased uptake of Slps by THP-1 DC may be important for the induction of IL-12 production in THP-1 DC. There were no significant differences in cytokine productions with bacterial cells collected at different growth times.

### 3.3. Slps from Various LAB Strains and Binding to DC-SIGN

**Figure 3:**
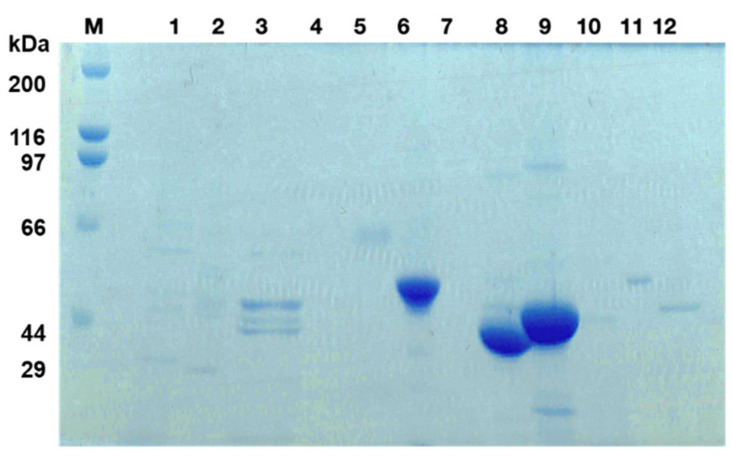
Sodium dodecyl sulphate-10% polyacrylamide gel electrophoresis (SDS-10%PAGE) analysis of 5 M LiCl released proteins. Lane 1: *L. plantarum* subsp. *plantarum* JCM 1149, lane 2: *L. lactic* subsp. *cremoris* NZ 9000, lane 3: *L. amylovorus* JCM 1126, lane 4: *L. paracasei* subsp. *paracasei* JCM 8130, lane 5: *L. kefiri* JCM 5818, lane 6: *L. brevis* JCM 1059, lane 7: *L. plantarum* JCM 1100, lane 8: *L. helveticus* JCM 1120, lane 9: *L. acidophilus* JCM 1132, lane 10: *L. plantarum* TIN- KL 001, lane 11: *L. lactis* subsp. *lactis* IL1403, lane 12: *L. rhamnosus* JCM 1136 and lane M: Marker proteins.

Previous studies have reported on the ability of the SlpA in the 5 M LiCl extract to bind the DC-SIGN receptor on THP-1 DC [21,30]. Thus far, no other Slps from the tested LAB strains have been described to have the ability to bind the DC-SIGN on THP-1 DC. Therefore, the Slps released in the 5 M LiCl extracts of various LAB strains were compared by SDS-10% PAGE analysis [27]. Expectedly, dense bands with molecular sizes of 47 and 45 kDa, corresponding to SlpA, were observed in *L. helveticus* JCM 1120 and *L. acidophilus* JCM 1132, respectively (Figure 3, lanes 8 and 9). In contrast, a major band with a molecular weight of 52 kDa was observed for *L. brevis* JCM 1059 (Figure 3, lane 6). The 52 kDa protein isolated from *L. brevis* JCM 1059 was identified as SlpB based on the proteome analysis after trypsin digestion of the excised gel by SDS-10%PAGE.

**Figure 4:**
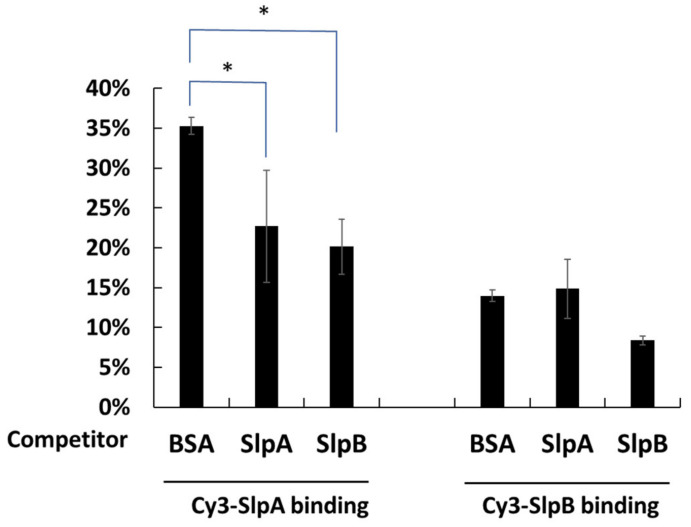
Bindings of ^Cy3^SlpA prepared from *L. acidophilus* JCM 1132 and ^Cy3^SlpB prepared from *L. brevis* JCM 1059 to THP-1 DC with or without 10 × non-labelled SlpA and SlpB. (Means ± SD. * *p* < 0.05).

DC-SIGN on THP-1 DC is known to be the receptor for SlpAs and is crucial for the cellular uptake of *L. helveticus* and *L. acidophils* [21,22,29]. Mincle is also known as the receptor for the Slps on *L. brevis* [24]. However, the receptor for SlpB, which is induced on THP-1 DC and is necessary for the binding of *L. brevis* JCM 1059, has not been elucidated thus far. To confirm the ability of SlpA of *L. acidophilus* JCM 1132 and SlpB of *L. brevis* JCM 1059 to share the DC-SIGN receptor, ^Cy3^SlpA and ^Cy3^SlpB were prepared for competitive binding assays. As shown in Figure 4, the binding of ^Cy3^SlpA SlpA to THP-1 DC was significantly inhibited by the addition of 10-fold of both, non-labelled SlpA and non-labelled SlpB. The binding of ^Cy3^SlpA SlpB showed a reduced trend (*p* = 0.069) with the addition of 10-fold non-labelled SlpB, but not with non-labelled SlpA. These results suggest that SlpB on *L. brevis* JCM 1059 binds to both, DC-SIGN and other receptors on THP-1 cells.

### 3.4. Purification and Identification of the Receptor for SlpB from THP-1 Cells

**Figure 5:**
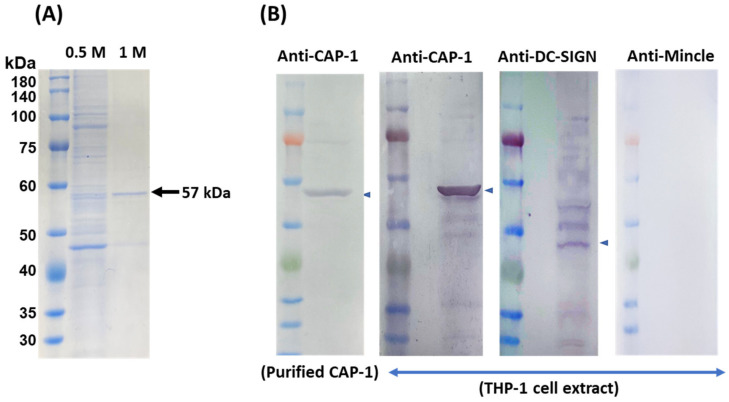
(**A**) SDS-10%PAGE of the affinity-purified 57 kDa receptor. (**B**) Western blotting analysis of affinity purified protein with anti-adenylyl cyclase-associated protein 1 (CAP-1) and crude extract of THP-1 cells with anti-CAP-1, anti-DC-SIGN, and anti-macrophage-inducible C-type lectin (Mincle) antibodies.

**Figure 6:**
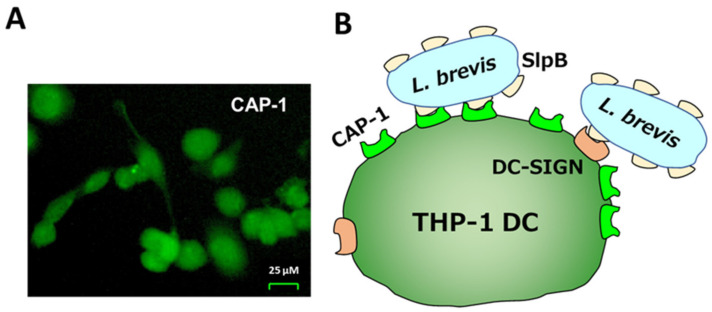
(**A**) Immunostaining of THP-1 DC with anti-CAP-1 antibody. CAP-1 on THP-1 DC was detected by incubation with FITC-labelled anti-CAP-1 antibody. (**B**) Illustration for *L. brevis* JCM 1059 binding of SlpB with CAP-1 and DC-SIGN.

Probable SlpB receptors in the THP-1 DC cell surface extract (THP-1 DC extract) were isolated with 0.1% Triton-PBS and applied to SlpB coupled Profinity Epoxide resins (SlpB-resin). The THP-1 DC extract was mixed with the SlpB-resin and the bound components were eluted with 0.5 and 1.0 M NaCl-PB after washing with 50 mM NaCl-PB. SDS-10%PAGE analysis showed the release of various sizes of components in 0.5 M elution, but a main protein with 57 kDa in 1 M NaCl-PB (Figure 5A). Proteome analysis indicated that the 57 kDa protein was CAP-1 [31,32]. To confirm the presence of CAP-1, and reported C-type lectin receptors, DC-SIGN, and Mincle in the THP-1 DC extract, western blotting was performed. A single band corresponding to 57 kDa was observed with anti-CAP-1 antibody for the affinity purified CAP-1 protein and for THP-1 cell extract (Figure 5A). The antibody against DC-SIGN showed a weak reaction towards some proteins including DC-SIGN corresponding reaction (arrow) as observed in previous study (29). However, no signal for Mincle was observed in THP-1 DC (Figure 5B). CAP-1 was observed on the whole cell surface of THP-1 DC when FITC-labelled anti-CAP-1 DC antibody was used (Figure 6A), as illustrated in Figure 6B.

### 3.5. Inhibition of SlpB Binding to THP-1 DC by Anti-CAP-1, Anti-DC-SIGN, and Anti-Mincle Antibodies

**Figure 7:**
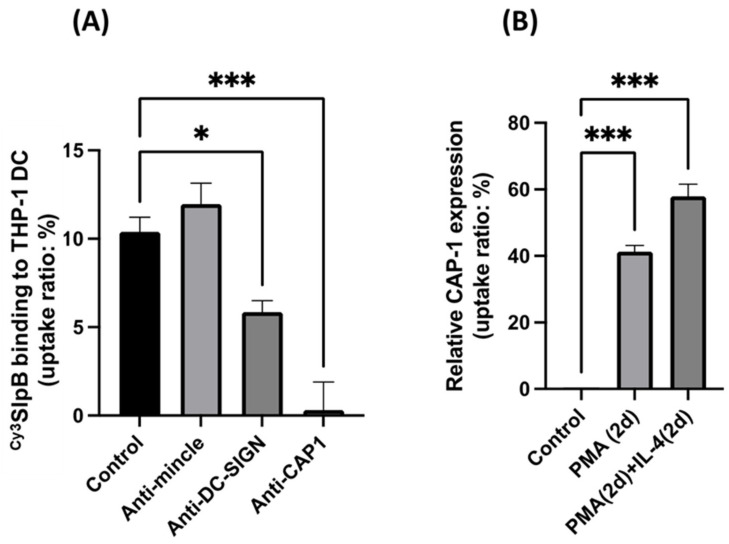
(**A**) ^Cy3^SlpB was incubated with THP-1 DC with normal mouse serum (Control), anti-Mincle antibody, anti-DC-SIGN antibody, and anti-CAP-1 antibody for 1 h. (**B**) CAP-1 expression in THP-1 DC differentiated with PMA, and PMA and IL-4 (**B**). (Means ± SD. * *p* < 0.05; *** *p* < 0.001).

To confirm the preferential binding of SlpB to the receptors on THP-1 DC, we monitored the ability of ^Cy3^SlpB to bind THP-1 DC in the presence of different antibodies. Addition of anti-CAP-1 antibody prevented the binding between ^Cy3^SlpB and THP-1 DC (Figure 7A). The anti-DC-SIGN antibody also caused significant inhibition of the interaction between ^Cy3^SlpB and THP-1 DC, whereas no significant inhibition was observed with the anti-Mincle antibody. The signal for DC-SIGN toward THP-1 DC extract was not strong in Western blotting (Figure 5); however, the specific binding of JCM 1059 with DC-SIGN was confirmed by the addition of anti-DC-SIGN antibody (Figure 7A). CAP-1 expression was induced in THP-1 DC after PMA and PMA/IL-4 treatment (Figure 7B).

**Figure 8:**
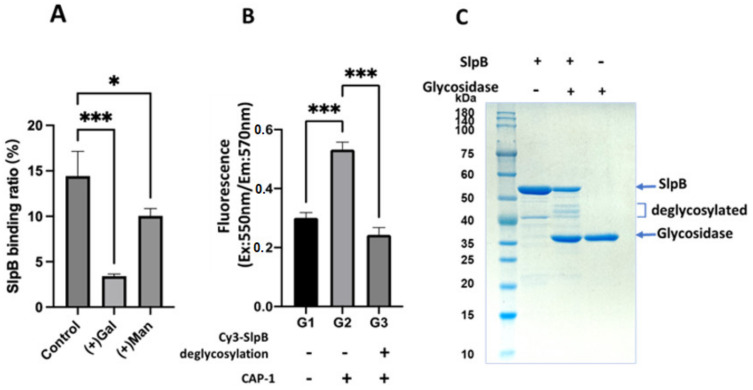
(**A**) ^Cy3^SlpB and THP-1 DC binding with or without galactose (Gal) and mannose (Man). (**B**) Binding between CAP-1 and ^Cy3^SlpB or glycosidase treated ^Cy3^SlpB. (**C**) SDS-10%PAGE analysis of SlpB and deglycosylated SlpB. (Means ± SD. * *p* < 0.05; *** *p* < 0.001).

DC-SIGN is known for its ability to bind the carbohydrates in SlpA, and mannose prevents SlpA-DC-SIGN binding [22]. Therefore, we evaluated the effect of mannose and galactose on SlpB-THP-1 DC binding. As shown in Figure 8A, mannose and galactose strongly inhibited SlpB binding. Moreover, the interaction between ^Cy3^SlpB and CAP-1 was significantly decreased after glycosidase treatment of ^Cy3^SlpB (Figure 8B). Miner bands likely released from SlpB were observed after glycosidase treatment (Figure 8C). These results suggested that CAP-1 can recognise the carbohydrates on SlpB and function as a lectin-like protein. To confirm the existence of carbohydrates on SlpB, CAP-1 was captured on a microplate with anti-CAP-1 antibody and thereafter, ^Cy3^SlpB was detected on the CAP-1 coated microplate. However, ^Cy3^SlpB was not captured when ^Cy3^SlpB was pre-treated with glycosidase (Figure 8B). These results indicate the presence of lectin-like activity of CAP-1 and are demonstrative of its interaction with the carbohydrates on SlpB.

### 3.6. Cytokine Production in THP-1 DC

**Figure 9:**
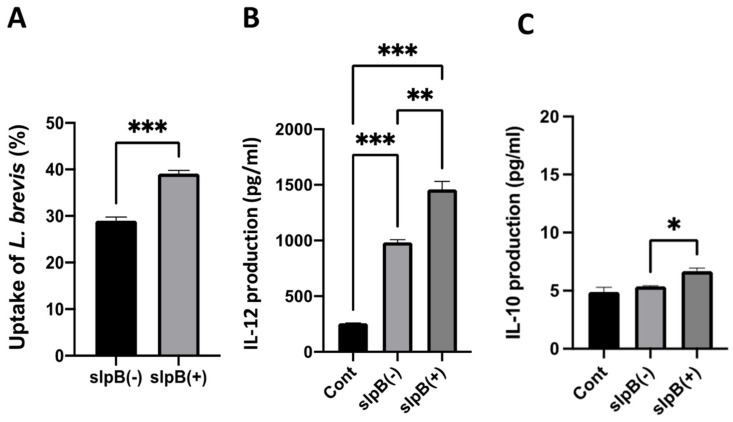
(**A**) Uptake of *L. brevis* JCM 1059 (slpB +) and LiCl treated JCM 1059 (slpB-) coating into THP-1 DC. (**B**) IL-12, and (**C**) IL-10 production by THP-1 DC after treatment with *L. brevis* with (+) or without (-) SlpB. (Means ± SD. * *p* < 0.05; ** *p* < 0.01, *** *p* < 0.001).

To evaluate the impact of the SlpB from *L. brevis* JCM 1059 on bacterial uptake by THP-1 DC, we used Slp (mainly SlpB) removed *L. brevis* JCM 1059 and Slp associated JCM 1059 to monitor bacterial uptake and cytokine production. The uptake of *L. brevis* JCM 1059 was significantly reduced when Slp was removed from the cell surface (Figure 9). Furthermore, Slp associated *L. brevis* JCM 1059 induced robust IL-12 production, whereas Slp removed *L. brevis* JCM 1059 showed significantly reduced IL-12 production. The levels of IL-10 and IL-6 were relatively low. However, Slp associated *L. brevis* JCM 1059 induced IL-10 and IL-6 production. These results suggest that binding of SlpB in Slp fraction to the receptors on THP-1 DC may trigger *L. brevis* JCM 1059 uptake and cytokine (especially IL-12) production (Figure 9) during 24 h incubation.

## 4. Discussion

Previous studies have highlighted the importance of the microbiota in the host immune system and other gut functions [33,34]. In the host gut immune system, little is known about the sampling of intestinal bacteria. Moreover, the Slps-receptor interactions between some *Lactobacillus* species, and DCs or macrophages facilitate bacterial uptake and induce the production of cytokines, such as IL-6, IL-10, IL-12, IL-17, and IL-23 [17,35,36]. Knocking out SlpA or the addition of a SlpA-specific antibody was shown to reduce the binding between *L. acidophilus* NCFM and THP-1 DC [21]. Furthermore, the interaction between the SlpA from *L. acidophilus* NCFM and DC-SIGN from THP-1 DC induced IL-10 and IL-12p70 production [21]. These results suggest that the frequent uptake of LAB by DC for 24 h may be essential for cytokine production. Most of previous studies mainly focused on C-type lectin receptor to identify receptors for SlpA [21] and SlpB [24] since C-type lectin localized on cell membrane of macrophage conserved carbohydrate recognition domains need for bacterial interactions [23]. In the present study, SlpB affinity purification was performed to screen SlpB receptors including non-C-type receptor and understand the role in the immunomodulatory responses and CAP-1 was identified as a novel SlpB receptor.

**Figure 10:**
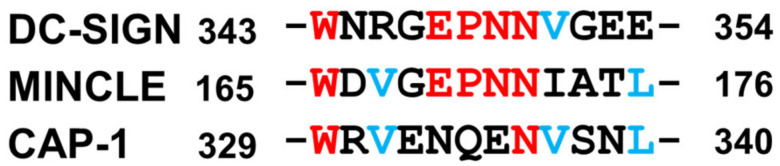
EPN sequence among DC-SIGN and Mincle, and similar sequence in CAP-1 Identical amino acids among 3 sequences were shown in red and between CAP-1 and Mincle of DC-SIGN were in blue.

In the present study, we observed that among the tested LAB strains, *L. helveticus* JCM 1120, *L. acidophilus* JCM 1132, and *L. brevis* JCM 1059 had the highest uptake ratios, and could promote the productions of the proinflammatory (IL-12) and anti-inflammatory (IL-10) cytokines (Figure 2). Consistent with previous studies on the other *L. helveticus* and *L. acidophilus* strains, we identified SlpA to be the major Slps in the 5 M LiCl extracts from *L. helveticus* JCM 1120 and *L. acidophilus* JCM 1132 [21,29]. Furthermore, the present comparative study with various LAB strains is the first to demonstrate the importance of SlpB-CAP-1 binding for *L. brevis* JCM 1059 uptake and subsequent cytokine production by THP-1 DC. Previous studies have reported on the SlpB-dependent uptake of *L. kefiri* via its 3interaction with the Mincle receptor on macrophages [36]. However, Mincle was not expressed on the PMA/IL-4 differentiated THP-1 DC in the present study (Figure 5). SlpB binding to THP-1 DC was significantly reduced after the addition of anti-DC-SIGN antibodies. However, the decrease in SlpB binding was higher with the anti-CAP-1 antibody than that with the anti-DC-SIGN antibody. The higher production of CAP-1 than that of DC-SIGN (Figure 5) could be the main reason for the frequent access to THP-1 DC. Although CAP-1 was involved in the SlpB-dependent uptake of *L. brevis* JCM 1059 by THP-1 DC, the mechanism of binding and specificity remain unclear. Previously, CAP-1 was reported as a functional receptor for resistin expressed on monocyte and activated intracellular signalling pathway to modulate NF-κB-related inflammatory cytokines [31]. DC-SIGN is a C-type lectin receptor that contains the carbohydrate-recognition domain called Glu-Pro-Asn (EPN) [37] and has the potential to bind glucose-, mannose-, and N-acetylglucosamine-containing oligosaccharides [38]. There was no clear EPN sequence in CAP-1 sequence, but was low homology with DC-SIGN and Mincle (EPN-like sequence in Figure 10). Both galactose and mannose reduced SlpB binding to THP-1 DC (Figure 8A), indicative of the involvement of the galactose and mannose from SlpB in its binding with CAP-1. However, the ability of DC-SIGN and CAP-1 to recognise the different carbohydrates on SlpB remains unknown.

DC-SIGN on THP-1 DC is crucial for its interaction with the SlpA on *L. helveticus* and *L. acidophilus* [21,30]. In the present study, we generated ^Cy3^SlpA and ^Cy3^SlpB to compare the ability of SlpB from *L. brevis* JCM 1059 and SlpA from *L. helveticus* JCM 1120 and *L. acidophilus* JCM1132 to interact with THP-1 DC. The binding between ^Cy3^SlpA and the receptors on THP-1 DC was significantly inhibited by the addition of excess amounts of non-labelled SlpA or SlpB (Figure 4 and Figure 7). In contrast, the interaction between ^Cy3^SlpB and THP-1 DC was replaced by non-labelled SlpB but not by SlpA (Figure 7). SlpB has low homology with SlpA but shares the receptor DC-SIGN on THP-1 DC with the protein. Previous studies have predicted the carbohydrate-binding module sequences in the SlpB from *L. kefiri* that are involved in recognising DC-SIGN [23]. Carbohydrate-binding module-like sequences were also observed in the SlpB from *L. brevis* JCM 1059 (Figure 10).

The present study is the first to show that the ability of CAP-1 to bind the SlpB from *L. brevis* 1059 was greater than that of DC-SIGN. *L. brevis* can activate THP-1 cells after SlpB-dependent uptake by CAP-1. This contributes to our present knowledge of the immunomodulatory effect of SlpB-positive LAB on the gut immune system especially on gut-associated lymphoid tissues (GALT) and the host response.

## 5. Conclusions

Comparative studies with different 12 LAB species for the cytokine productions and the cell surface proteins revealed that SlpA on *L. helveticus* JCM 1120 and *L. acidophilus* JCM 1132 plays a crucial role for bacterial uptake by THP-1 DC. In contrast, SlpB on *L. brevis* JCM 1059 was a crucial to bind to THP-1 DC and following proinflammatory cytokine IL-12 production. SlpB receptor on THP-1 DC was purified by SlpB coated affinity resin and identified as CAP-1. CAP-1 expression on THP-1 DC was higher than that of DC-SIGN reported as the receptor for SlpB in WB analysis. Moreover, SlpB binding to THP-1 DC was completely inhibited by adding of anti-CAP-1 antibody and deglycosylation of SlpB, suggesting CAP-1 with interaction with carbohydrates on SlpB might be a major receptor for SlpB on THP-1 DC. Here, we identify a novel SlpB receptor CAP-1 on THP-1 DC which plays a crucial role in immunomodulatory effect of *L. brevis* in THP-1 cells.

## Figures and Tables

**Table 1:** Lactic acid bacteria and culture incubation temperature.

Strain	Temperature (°C)	Origin
*Lactobacillus helveticus* JCM 1120	37	Cheese
*Lactobacillus acidophilus* JCM 1132	37	Human feces
*Lactobacillus amylovorus* JCM 1126	37	Cattle waste
*Lacticaseibacillus rhamnosus* JCM 1136	37	Unknown
*Lactobacillus delbrueckii* subsp. *bulgaricus* JCM 1002	37	Bulgarian yogurt
*Levilactobacillus brevis* JCM 1059	30	Human feces
*Lactiplantibacillus plantarum* JCM 1100	30	Unknown
*Lentilactobacillus kefiri* JCM 5818	30	Kefir grains
*Lacticaseibacillus paracasei* subsp. *paracasei* JCM 8130	30	Milk product
*Lactiplantibacillus plantarum* subsp. *plantarum* JCM 1149	30	Pickled cabbage
*Lactococcus lactis* subsp. *cremoris* NZ9000	30	Mutant of *L. lactis* MG1363
*Lactococcus lactis* subsp. *lactis* IL1403	30	Cheese

All LAB strains obtained from the Japan Collection of Microorganisms (JCM) and our culture collections were cultured in MRS medium at 30 °C or 37 °C for 20 h. Reported origin for each strain was showed.

## Data Availability

Not applicable.
